# Single and Concurrent Effects of Endurance and Resistance Training on Pulmonary Function

**Published:** 2013-04

**Authors:** Maryam Khosravi, Seyed Morteza Tayebi, Hamed Safari

**Affiliations:** 1 Exercise Physiology Division, Faculty of Physical Education and Sport Science, Islamic Azad University-Ayatollah Amoli Branch, Amol, Mazandaran, Iran; 2 Faculty of Physical Education and Sport Science, Science and Research Branch, Islamic Azad University, Hamedan, Iran

**Keywords:** Endurance and Resistance Training, FEV1, FVC, MVV, PEF, VC

## Abstract

***Objective(s):*** As not only few evidences but also contradictory results exist with regard to the effects of resistance training (RT) and resistance plus endurance training (ERT) on respiratory system, so the purpose of this research was therefore to study single and concurrent effects of endurance and resistance training on pulmonary function.

***Materials and Methods:*** Thirty seven volunteer healthy inactive women were randomly divided into 4 groups: without training as control (C), Endurance Training (ET), RT, and ERT. A spirometry test was taken 24 hrs before and after the training course. The training period (8 weeks, 3 sessions/week) for ET was 20-26 min/session running with 60-80% maximum heart rate (HR max); for RT two circuits/session, 40-60s for each exercise with 60-80% one repetition maximum (1RM), and 1 and 3 minutes active rest between exercises and circuits respectively; and for ERT was in agreement with either ET or RT protocols, but the times of running and circuits were half of ET and RT.

***Results:*** ANCOVA showed that ET and ERT increased significantly (*P<* 0.05) vital capacity (VC), forced vital capacity (FVC), and forced expiratory flows to 25%-75%; ET, RT and ERT increased significantly (*P<* 0.05) maximum voluntary ventilation (MVV); and only ET increased significantly (*P<*0.05) peak expiratory flows (PEF); but ET, RT and ERT had no significant effect (*P>*0.05) on forced expiratory volume in one second (FEV1) and FEV1/FVC ratio.

***Conclusion:*** In conclusion, ET combined with RT (ERT) has greater effect on VC, FVC, FEF rating at25%-75%, and also on PEF except MVV, rather than RT, and just ET has greater effect rather than ERT.

## Introduction

Efficacy of respiratory and pulmonary functions has a direct relationship with general health (1). Furthermore, regular physical activity is of much importance for general health of people, especially young people (2, 3). Since cardiorespiratory endurance is a key component of physical fitness and physical activity can lead to physical fitness, so it can improve cardiorespiratory endurance (4). It is well-documented that the most effective factor in cardiorespiratory fitness is physical activity level (5-7). Exercise training improves endurance and strength of athletes’ respiratory muscles; it also causes resistance reduction in respiratory canals, and increases lung elasticity and alveolar expansion as studies have supported the expansion of pulmonary volumes and capacities (8). Accordingly, selection of appropriate type of exercise training may be an important factor in prevention or decrease of respiratory diseases and increase the efficacy of this system. 

It has been proved that the inability to maintain ventilation with high levels is a factor for restricting maximal aerobic capacity in healthy people (9-11). Although some evidences have reported that the pulmonary system is un- affected by physical activity (12, 13), Crosbie, (2012) after a systematic review of randomized control trials (16 studies and 516 subjects met inclusion criteria), suggested that physical training increases aerobic capacity measured by VO_2max_, and didn’t enhance pulmonary function in children with asthma (14); but it is mainly recommended in various researches that endurance training (ET) is appropriate for improvement of pulmonary function (15); and others found that body endurance is increased by respiratory trainings (16). On the other hand, the effects of RT on inspiratory and expiratory muscles has been investigated and it was concluded that they have positive impacts, both on healthy people and individuals with chronic obstructive pulmonary disease (15, 17, 18). But recently parallel effects of ET and resistance training (RT) have been considered since the studies show further beneficial effects of strength training on improvement of ET function (19). There are some evidences related to RT (15) and parallel training effects on pulmonary function; these studies were done on patients with chronic obstructive pulmonary disease and showed a positive impact on pulmonary function indices (20, 21), but little study has been done on healthy individuals regarding the effect of inspiratory (IMT) (18, 22) and concurrent respiratory muscle training (CRMT) (17) [which are approximately equal to training regimens used in systemic exercise (22)] on pulmonary function and contradictory results have been reported different studies. 

Besides, it is known that Iranian women have poor situations for active life style because of their religious beliefs. They might be at risk even if they are healthy. Considering the little evidence about the effect of RT and also endurance plus resistance training (ERT) (23) except healthy subjects specially healthy inactive women, as well as contradictory results of these reports, we aimed to investigate the parallel effects of ET and RT on pulmonary function of healthy inactive women. Therefore we selected four groups with three types of training and tried to keep the intensity and duration of these three types of training the same.

## Materials and Methods

*Subjects*

The study was approved by the research ethics committee of the School of Medical Sciences of Islamic Azad University (Iran), and conducted in accordance with policy statement of the Declaration of Iranian Ministry of Health. Written informed consent was obtained from young inactive healthy women (20-35 years old) who registered in “Hirboud Sport Club” at recent summer (n = 85). All subjects were asked to complete a medical examination as well as a medical questionnaire to ensure that they were not taking any regular medications, and were free of cardiac, respiratory, allergic, eye and ear surgery, respiratory epidemic infections, uncontrolled blood pressure, thorax surgery history in three weeks before beginning trainings, history of pulmonary embolism, active hemoptysis, unstable angina, or myocardial infarction. Then, the acceptable volunteers (n = 36) were classified randomly into 4 groups as follows: a control group (n=9) without training (C), ET group (n=9), RT group (n=10), and ERT group (n=9).

*Research design*

Participants were taken to the practice hall two times before the beginning of training period. In the first session, their one repeat maximum (1RM) was determined for each of 8 exercises (bench press, curl up, arm extension, leg press, knee flexion, knee extension, plantar flexion, and sit up). In the second session, a spirometry test was performed on each participants (spirometer: Spiro lab, SN: A23-050-7460, Mir Co, Italia) for VC, FVC, FEF 25%-75%, FEV1, FEV1/FVC ratio, PEF, and MVV measurements. Also, HR_max_ was calculated (HR_max_ = 220 - age) for each one and controlled with heartbeat determinant girdle (Phase, Germany) during training. The training sessions started 24 hrs after the spirometry test lasting for 8 weeks, that is three days a week (Saturday, Monday, and Wednesday). 

*Exercise training procedures*

The subjects were instructed to follow a normal lifestyle, to maintain daily habits, and to avoid any regular medications. Each session contained 10 min-warm up and cool down. ET runs for 20 min/sessions with 60-65% HR_max_ in the first 4 weeks and 26 min/session with 65-80% HR_max_ in the second 4 weeks. RT training included two circuits/sessions, 60s (about 12 repeats) for each exercise with 60-65% 1RM, and 1 and 3 minutes specified to active rest between exercises and circuits respectively during the first 4 weeks; and four circuits/session, 40s (about 8 repeats) for each exercise with 65-80% 1RM and same rest periods during the second 4 weeks. ERT exercises were in agreement with either ET or RT protocols, but the times of running and circuits was half of ET and RT, respectively (Table 1).

**Table 1:** Summary of Exercise Training Procedures

Types of Training	four weeks	Intensity	Volume	Each exercise for ET	Rest for RT
ET	1st	60-65% HRmax	20min/session		
	2nd	65-80% HRmax	26min/session		
RT	1st	60-65% 1RM	2circuits/session	60s(12repeats)	1 min between exercises and 3min between circuits
	2nd	65-80% 1RM	4circuits/session	40s(8repeats)	
ERT	1st	60-65% HR_max_ plus 60-65% 1RM	10min/session plus 1circuits/session	60s(12repeats)	
	2nd	65-80% HR_max_ plus 65-80% 1RM	13min/session plus 2circuits/session	40s(8repeats)	

ET: Endurance Training; RT: Resistance Training; ERT: Endurance plus Resistance Training

*Statistics*

We used ANCOVA to determine the effects of the mentioned three types of training at significance levels of *P=* 0.05. Important ANCOVA assumptions, including linear relationship of dependent variable and covariate, normal distribution, and equality of error variances were examined by Pearson’s correlation test, one-sample Kolmogorov-Smirnov test, and Levene test, respectively. In variables that assumptions of normal distribution and equality of error variances were not met, we first subtracted dependent variables (post-test) by covariates (pre-test) as new dependent variables. Thus the effects of covariates were removed; then we used Kruskal-Wallis test for k-independent samples, and Mann-Whitney test for paired comparisons as post-hoc test. Significance levels in these paired comparisons was *P=* 0.0083 (Bonferroni adjustment for multiple comparisons).

## Results

General data of subjects including age, and body mass index (BMI) are summarized in Table 2.

Assumptions of linear relationships and normal distribution were met in all variables; however, the one assuming equality of error variances was met only in VC (F = 1.98, *P=* 0.13), FVC (F = 2.69, *P=* 0.061), MVV (F = 0.69, *P=* 0.56), and PEF (F = 1.49, *P=* 0.235), but not in FEF 25%-75% (F = 5.93, *P=* 0.002), FEV1 (F = 3.31, *P=* 0.031), and FEV1/ FVC (F = 3.31, *P=* 0.031). 

**Table 2:** Descriptive statistics of subjects in four training groups (Mean ± SE)

Factors Types of Training	Age *(Year)*	BMI *(kg/m*2*)*
Endurance	28.55 ± 1.59	23.29 ± 0.94
Resistance	26.4 ± 1.22	22.26 ± 0.88
Endurance plus Resistance	27.44 ± 1.37	21.55 ± 0.47
Control	26.55 ± 0.94	24.44 ± 1.02

In ANCOVA results, the adjusted mean of dependent variables (post-test in this study) are presented by omission of covariate (pre-test in this study) effects.

**Figure 1:**
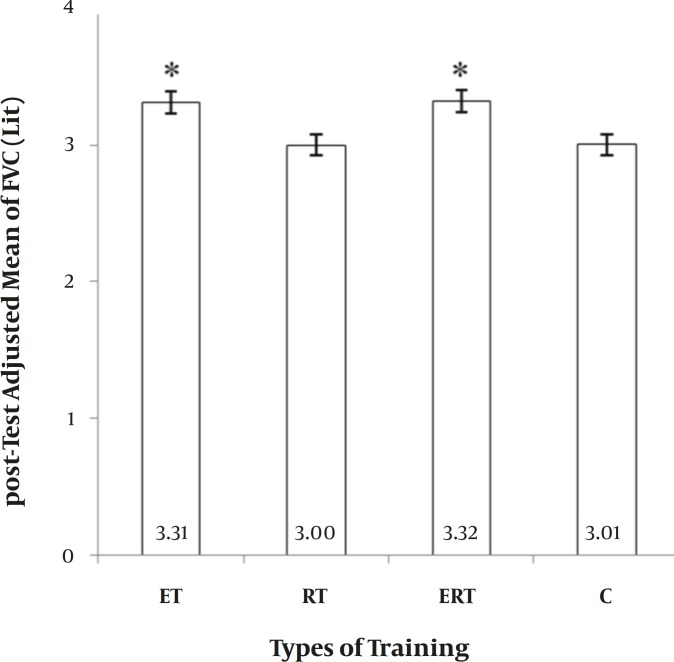
Between-group differences for Vital Capacity (VC). ET: endurance training. RT: resistance training. ERT: endurance plus resistance training. C: control without training. *: significantly greater than Resistance and Control group (P≤ 0.05).

**VC. **The main effect of “Group” on VC was significant (F = 5.201, *P=* 0.005). With respect to adjusted mean, ET and also ERT were significantly greater than RT and C. Other comparisons were not significant (Figure 1). It showed a 14.9%, 2.4%, 14.4%, and 3.4% elevation in ET, RT, ERT, and C, respectively. In other words, ET and ERT caused significant improvement in VC, but RT had no significant effect.

**Figure 2:**
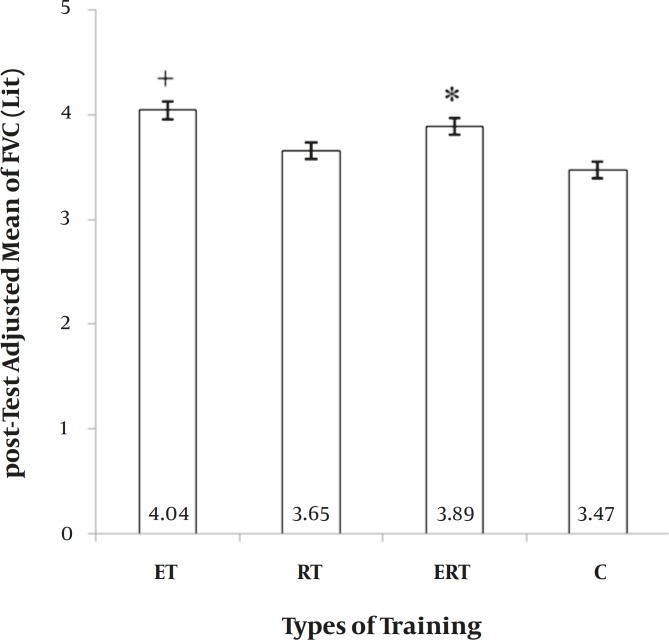
*Between-group differences for Forced Vital Capacity (FVC)*. ET: endurance training. RT: resistance training. ERT: endurance plus resistance training. C: control without training. +: significantly greater than Resistance and Control group (p ≤ 0.05). *: significantly greater than Control group (p ≤ 0.05).

**FVC**. The main effect of “Group” on FVC was also significant (F = 9.235, *P=* 0.000). ET was significantly greater than RT and C, and ERT was greater than C. Other comparisons were insignificant (Figure 2). It showed a 23.1%, 6.55%, 19.1%, and 3.2% increase in ET, RT, ERT, and C.

**Figure 3:**
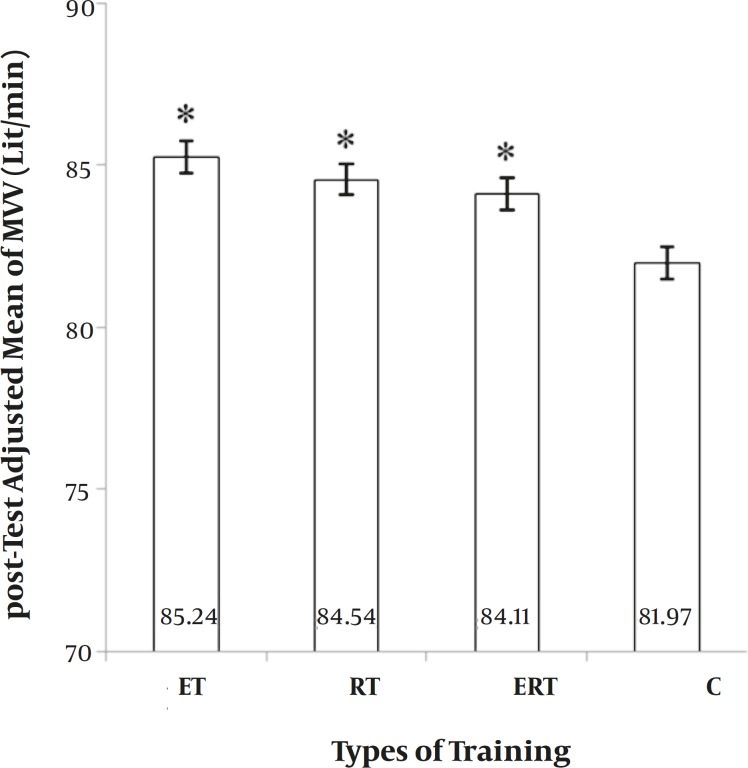
*Between-group differences for Maximum Voluntary Ventilation (MVV).* ET: endurance training. RT: resistance training. ERT: endurance plus resistance training. C: control without training. *: significantly greater than Control group (p ≤ 0.05).

**MVV.** The main effect of “Group” on MVV was also significant (F = 9.235, *P=* 0.000). ET, RT and their combination (ERT) were significantly greater than C; but other comparisons were not significant (Figure 3). It showed a 3.9%, 2.61%, and 2.45% elevation in ET, RT, and ERT, respectively, and a 0.05% depression in C.

**Figure 4:**
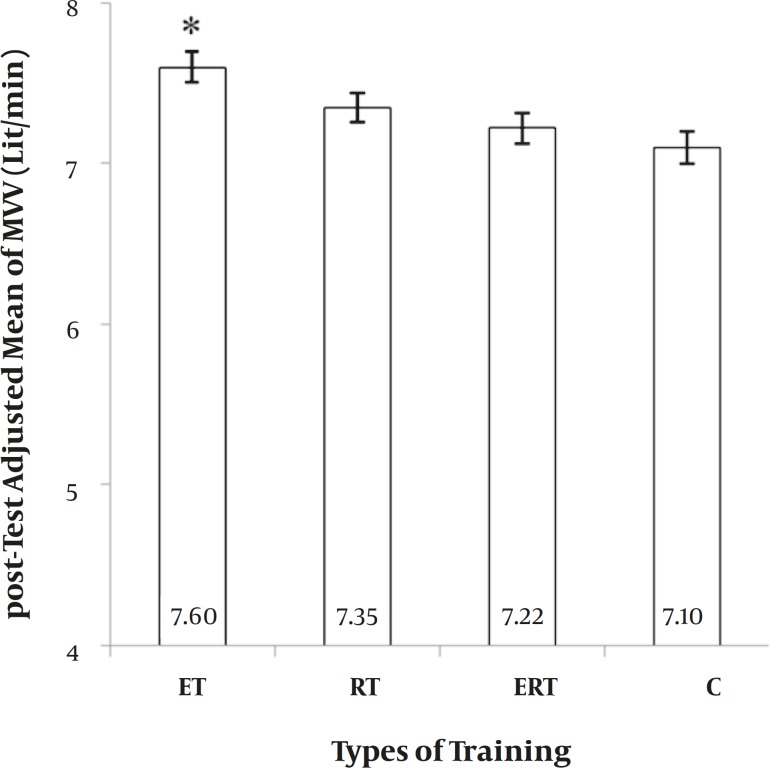
*Between-group differences for Peak Expiratory Flows (PEF).* ET: endurance training. RT: resistance training. ERT: endurance plus resistance training. C: control without training. *: significantly greater than Control group (p ≤ 0.05).

**Figure 5:**
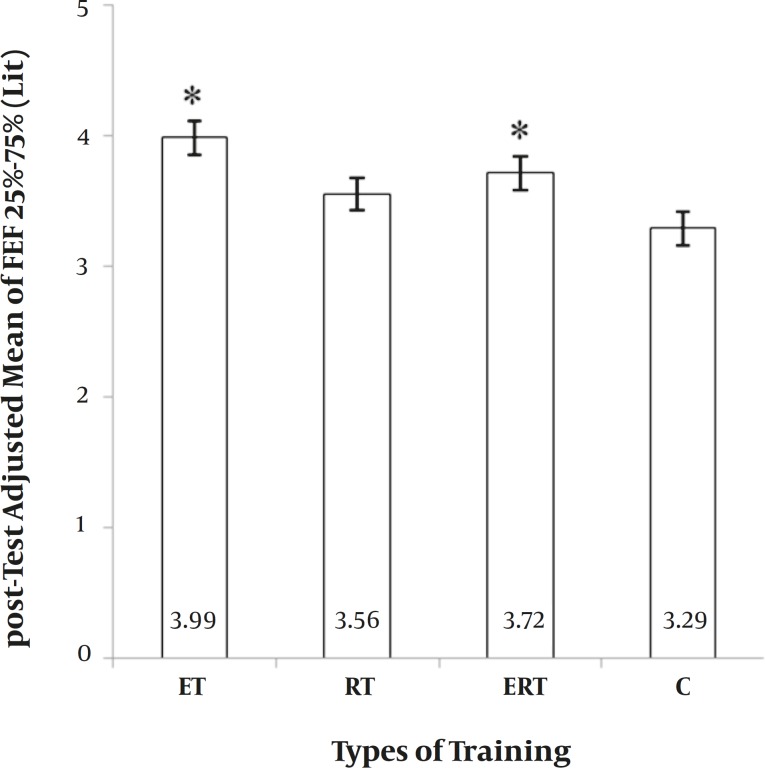
*Between-group differences for Forced Expiratory Flows (FEF25%-75%).* ET: endurance training. RT: resistance training. ERT: endurance plus resistance training. C: control without training. *: significantly greater than Control group (p ≤ 0.05).

**PEF.** The main effect of “Group” on PEF was also significant (F = 4.966, *P=* 0.006). ET was significantly greater than C, but other comparisons were not significant (Figure 4). It showed a 8.5%, 5.7%, 4.0%, and 2.04% elevation in ET, RT, ERT and C respectively.

**FEF 25%-75%.** Inter-group differences for FEF (25%-75%) were significant (Chi-square = 17.41, *P=* 0.001). Multiple comparisons showed that ET and ERT were significantly greater than control group. Other comparisons were not significant (Fig. 5). It showed a 25.1%, 8.6%, 14.1%, and 2.2% elevation in ET, RT, ERT and C respectively.

**FEV1** and **FEV1/FVC.** Inter group differences for FEV1 and FEV1/FVC were not significant [(Chi-square = 7.55, *P=* 0.056) and (Chi-square = 2.327, *P=* 0.507), respectively] (Table 3). FEV1 showed a 10.7%, 10.4%, 5.6%, and 2.8% elevation in ET, RT, ERT and C respectively; and, FEV1/FVC was increased for 1.45%, 0.8%, and 0.6%, in ET, RT, and ERT respectively and was decreased for 0.14% in C.

Discussion

In this study, the VC (equal to sum of expiratory reserve volume, inspiratory reserve volume, and flowing volume) in ET and ERT was significantly greater than that in RT and C, which was found after 8 weeks of training as compared to the baseline. On the other hand, the obtained results indicate that significant improvement in VC of ERT may be due to the effects of endurance training which are summed with the effects of resistance training. Also, the FVC, i.e. maximum amount of air exited from lung with a deep expiration after a deep inspiration, was significantly greater in ET than in RT and C, and was significantly greater in ERT than in C, which was found after 8 weeks of training as compared to the baseline. In other words, ET and ERT caused significant improvement in FVC, but RT had no significant effect. As support of our results, researches have shown that active persons (24) and athletes (25) have a higher level of VC and FVC compared to healthy control group. Nourry *et al *(2005) reported a significant increase in FVC of healthy prepubescent children after 8 weeks of high-intensity intermittent running training (26). Farid *et al* (2005) found significant changes in FVC after eight weeks of aerobic exercise (asthmatic patients, 3sessions/week, 20min/session, 15min warm up and tensile exercise before main exercise/session) (27). Tartibian, Maleki, & Abbasi. (2010) found a significant increase in VC and FVC after wrestling training (12 weeks, 3times/week, 90–120 min/day to 70–85% of HR_max_ in the first 6 weeks, and to 85–95% of HR_max_ in the second 6 weeks) (28). Enright and Unnithan. (2011) found an increase in VC after 8-week IMT program (80% of sustained maximum inspiratory effort, 3times/week inconsecutively) (22). Shaw, , and Brown. (2011), after 16 weeks aerobic (45min, 60%HR_max_), resistance (8 exercises, 60%1RM, 3 sets, 15 repetitions) and concurrent training, found that both aerobic and concurrent training had an effect on improvement of some pulmonary functions, but concurrent training had the most effect on improvement of FVC, FEV(1), PEF, and FEF 25%-75% (23) in sedentary male smokers.

In our study, the expansion of VC and FVC can be due to the high-rate ventilation during ET gravely requesting the respiratory muscles. However, FVC is usually lower than VC (29). Therefore, the increase in FVC could result from an enhancement in contractility of expiratory muscle without real change in lung volume (30). But we observed a significant increase in VC. 

Moreover, the MVV (total volume of air replaced with inspiration and expiration in maximum depth and speed) in ET, RT, and ERT was significantly greater than C after 8 weeks of training as compared to the baseline. In other words, ET, RT and ERT caused significant improvement in MVV. Farid *et al* (2005) reported that there was significant expansion in MVV (27). Tartibian *et al *(2010) also showed a significant increase in MVV (28). Galvan, & Cataneo. (2007) believe that MVV can indicate the function of only respiratory muscles and therefore, the values of MVV increase on account of improved strength of inspiratory muscles (31). But we know that MVV is maximum voluntary ventilation and not only inspiratory muscles but also expiratory muscles are involved in voluntary ventilation with strength; so depending on the force -generating capacity of inspiratory and expiratory muscles, strength of these muscles is improved during 8 weeks of ET, RT and ERT.

In addition, the PEF (maximum volume of air flow during a deep expiration maneuver) in ET, RT and ERT was significantly greater than that in C after 8 weeks of training as compared to the baseline. In other words, ET caused significant improvement in PEF as compared to other types of training. Nourry *et al *(2005) observed a significantly increased PEF after running training (26). Also, Farid *et al *(2005) found a significant expansion in PEF after eight weeks of aerobic exercise in asthmatic patients (27). 

Furthermore, the FEF 25%-75% (volume of air flow exiting the lung during mid time of FVC) in ET, and ERT, toward RT was significantly greater than that in C after 8 weeks of training as compared to the baseline. In other words, ET and ERT caused significant improvement in FEF (25%-75%) as compared to RT. Farid *et al *(2005) and Tartibian *et al *(2010) both also found the significant increase in FEF (25-75%) after exercise training (27, 28).

Nevertheless, intergroup differences for FEV1 (volume in one second is volume of air that exits the lung in 1-s of a deep and potent expiration) and FEV1/FVC or the ratio of forced expiratory volume in 1-s to forced vital capacity (i.e. the percentage of FVC which exits the lung during first second of a deep expiration) were not significant after 8 weeks of training as compared to the baseline, especially to C; that is, each type of training could not significantly affect FEV1 and FEV1/FVC.

Grisbrook *et al* (2012), after a 12 - week goal directed interval and resistance training didn’t find any change in FEV1/FVC ratio of adults with burn (32). Cheng *et al *(2003) showed that active persons had a higher FEV1 than the others (24). Besides, Wright *et al *(2003) after a hypertrophic maximal strength training [12 weeks (2 weeks of muscle habituation training, 5 weeks of hypertrophic training I, 5 weeks of hypertrophic training II with intensified eccentric work), initially twice, then 3 times a week, 60-120 min] in patients with chronic obstructive pulmonary disease (COPD) observed that FEV1 is elevated (5.3%) significantly compared to the baseline (15). Nourry *et al *(2005) and Tartibian, Maleki, & Abbasi. (2010) reported a significant increase in FEV1 after training (26). Farid *et al *(2005) also found that there was a significant rise in FEV1, but FEV1/FVC showed no significant change (27).

Based on a literature, Nourry *et al *(2005) (26) interpreted that the improvement in expiratory flow parameters such as PEF, FEF (25%-75%), FEV1, and FEV1/FVC could be illustrated by one or two causes: an increase in contractility (33) or strength (34, 35) of the expiratory muscle, or alterations in the lung compliance and the balance in airway resistance (34, 36), as we described in introduction. 

However description of our results must remain speculative, as it is possible that these types of training cause the deterioration of active and passive movement systems, which are caused by inactivity-specific deconditioning processes. As Tartibian, *et al* (2010) concluded, these variables were influenced by exercise training because in inactive control group they showed a significant decrease following 12 weeks of detraining (28). Promotion of skeletal muscles’ endurance is related to the increase in their oxidative capacity through the enhancement in levels of oxidative enzymes, reserves of lipids and glycogen, and the number of capillaries; however, development of strength is related to increase in synthesis of the contractile proteins (actin and myosin) due to the long-term training programs. Various studies mention that the capacity of respiratory muscles can be elevated through appropriate stimuli that so that raises their workload, and therefore the training of respiratory muscles through multiple types of exercise training purposes to expedite these kinds of cellular changes in the activated muscles (31).

Multiple aspects may support improvement of the pulmonary function. We know that muscular imbalance-associated inactivity causes a restriction in the thorax (15) and so exercise training may have a compensatory effect on this situation; furthermore, reinforcement of the auxiliary respiratory muscle is another effect of regular exercise training (37). It has been shown in previous studies on asthmatic patients that physical exercise can increase the residual air flow and decrease the ventilation by reinforcement of bronchi expansion during an exercise. This makes an asthmatic patient save air flow during exercise (27, 38). In addition, improved pulmonary function following exercise training could be due to decreased airway resistance, increased airway caliber, and strengthened respiratory muscles as well as lung and thorax elasticity (8, 28). On the other hand, hormonal effects, compromised roles of adrenaline (39, 40) and cortisol (41) also seem to be feasible. A decreased lung retractability and induced vasodilatation of pulmonary vessels are reported to be due to an increased activation of adrenaline system during exercise training in which vasodilatation of pulmonary vessels cause a decreased airway resistance and an enhanced FEV1 and FVC through increasing airflow (39, 40). Serum cortisol has also been reported to have a connection to bronchodilatation and lung surfactant generation (41).

## Conclusion

This study showed that a period of ERT caused a clear increase in VC, FVC, MVV, and FEF (25%-75%) as did ET. Also, PEF is elevated only due to ET; and RT improved only MVV as did ET and ERT in healthy young inactive women. FEV1 and FEV1/FVC were not affected significantly by any types of training. Results of our study may suggest that the type of training is related to the improvement of pulmonary function; however ET, RT and ERT have no difference in influence on VC, MVV, FEF 25%-75%, PEF, FEV1 and FEV1/FVC, but because ET caused an increase in VC, FVC, MVV, FEF 25%-75% and PEF, and RT added to ET (ERT) caused an increase in VC, FVC, MVV, FEF 25%-75%, we can conclude that ET has probably higher effects on some pulmonary function of healthy young inactive women as compared to not only RT but also to ERT.

## References

[1] WHO (2007). Promoting Physical Activity in Schools: An Important Element of a Health-Promoting School. WHO Information Series on School Health, Document Twelve. http://whqlibdoc.who.int/publications/2007/9789241595995_eng.pdf.

[2] Boreham C, Riddoch C (2001). The physical activity, fitness and health of children. J Sports Sci.

[3] Boreham CAG, Riddoch CJ, McKenna J, Riddoch CJ (2003). Physical activity and health through the lifespan. Perspectives in Health and Exercise.

[4] Caspersen CJ, Powell KE, Christenson GM (1985). Physical activity, exercise, and physical fitness: definitions and distinctions for health-related research. Public Health Rep.

[5] McCord P, Nichols J, Patterson P (1989). The effect of low impact dance training on aerobic capacity, submaximal heart rates and body composition of college-aged females. J Sports Med Phys Fitness.

[6] Maaroos J, Land A (2001). Anthropometric indices and physical fitness in university undergraduates with different physical activity. Anthropol Anz.

[7] Williams CL, Hayman LL, Daniels SR, Robinson TN, Steinberger J, Paridon S (2002). Cardiovascular health in childhood: a statement for health professionals from the committee on atherosclerosis, hypertension, and obesity in the young (AHOY) of the council on cardiovascular disease in the young, American Heart Association. Circulation.

[8] Leith DE, Bradley M (1976). Ventilatory muscle strength and endurance training. J Appl Physiol.

[9] Taylor BJ, Romer LM (2008). Effect of expiratory muscle fatigue on exercise tolerance and locomotor muscle fatigue in healthy humans. J Appl Physiol.

[10] Sheel AW, Derchak PA, Pegelow DF, Dempsey JA (2002). Threshold effects of respiratory muscle work on limb vascular resistance. Am J Physiol Heart Circ Physiol.

[11] Dempsey JA, McKenzie DC, Haverkamp HC, Eldridge MW (2008). Update in the understanding of respiratory limitations to exercise performance in fit, active adults. Chest.

[12] Hagberg JM, Yerg JE, Seals DR (1988). Pulmonary function in young and older athletes and untrained men. J Appl Physiol.

[13] Womack CJ, Harris DL, Katzel LI, Hagberg JM, Bleecker ER, Goldberg AP (2000). Weight Loss, Not Aerobic Exercise, Improves Pulmonary Function in Older Obese Men. J Gerontol A Biol Sci Med Sci.

[14] Crosbie A (2012). The effect of physical training in children with asthma on pulmonary function, aerobic capacity and health-related quality of life: a systematic review of randomized control trials. Pediatr Exerc Sci.

[15] Wright P, Heck H, Langenkamp H (2003). Effects of a resistance training on pulmonary function and performance measurements in patients with chronic obstructive pulmonary disease. Eur J Sport Sci.

[16] Pringle EM, Latin RW, Berg K (2005). The relationship between 10 km running performance and pulmonary function. J Exerc Physiol.

[17] Wells GD, Plyley M, Thomas S, Goodman L, Duffin J (2005). Effects of concurrent inspiratory and expiratory muscle training on respiratory and exercise performance in competitive swimmers. Eur J Appl Physiol.

[18] Enright SJ, Unnithan VB, Heward C, Withnall L, Davies DH (2006). Effect of high-intensity inspiratory muscle training on lung volumes, diaphragm thickness, and exercise capacity in subjects who Are healthy. Phys Ther.

[19] Chtara M, Chamari K, Chaouachi M, Chaouachi A, Koubaa D, Feki Y (2005). Effects of intra-session concurrent endurance and strength training sequence on aerobic performance and capacity. Br J Sports Med.

[20] Spruit MA, Gosselink R, Troosters T, De Paepe K, Decramer M (2002). Resistance versus endurance training in patients with COPD and peripheral muscle weakness. Eur Respir J.

[21] Mador MJ, Bozkanat E, Aggarwal A, Shaffer M, Kufel TJ (2004). Endurance and strength training in patients With COPD. Chest.

[22] Enright SJ, Unnithan VB (2011). Effect of inspiratory muscle training intensities on pulmonary function and work capacity in people who are healthy:a randomized controlled trial. Phys Ther.

[23] Shaw I, Shaw BS, Brown GA (2011). Concurrent training and pulmonary function in smokers. Int J Sports Med.

[24] Cheng YJ, Macera CA, Addy CL, Sy FS, Wieland D, Blair SN (2003). Effects of physical activity on exercise tests and respiratory function. Br J Sports Med.

[25] Mickleborough TD, Murray RL, Ionescu AA, Lindley MR (2003). Fish oil supplementation reduces severity of exercise-induced bronchoconstriction in elite athletes. Am J Respir Crit Care Med.

[26] Nourry C, Deruelle F, Guinhouya C, Baquet G, Fabre C, Bart F (2005). High-intensity intermittent running training improves pulmonary function and alters exercise breathing pattern in children. Eur J Appl Physiol.

[27] Farid R, Azad FJ, Atri AE, Rahimi MB, Khaledan A, Talaei-Khoei M (2005). Effect of aerobic exercise training on pulmonary function and tolerance of activity in asthmatic patients. Iran J Allergy, Asthma Immunol.

[28] Tartibian B, Maleki BH, Abbasi A (2010). The effects of omega-3 supplementation on pulmonary function of young wrestlers during intensive training. J Sci Med Sports.

[29] Préfaut C, Peslin R (1986). Breath tests or measurement of lung volume and bronchial flow. Rev Mal Respir.

[30] Koch G, Eriksson BO (1973). Effect of physical training on anatomical R-L shunt at rest and pulmonary diffusing capacity during near-maximal exercise in boys 11-13 years old. Scan J Clin Lab Invest.

[31] Galvan CCR, Cataneo AnJM (2007). Effect of respiratory muscle training on pulmonary function in preoperative preparation of tobacco smokers. Acta Cir Bras.

[32] Grisbrook TL, Wallman KE, Elliott CM, Wood FM, Edgar DW, Reid SL (2012). The effect of exercise training on pulmonary function and aerobic capacity in adults with burn. Burns.

[33] Farrell PA (1981). Maximal expiratory flow-volume relationships before and after eight weeks of endurance training. J Sports Med Phys Fitness.

[34] Clanton TL, Dixon GF, Drake J, Gadek JE (1987). Effects of swim training on lung volumes and inspiratory muscle conditioning. J Appl Physiol.

[35] EngströM I, Eriksson BO, Karluerg P, Saltin B, Thoren C (1971). Preliminary report on the development of lung volumes in young girl swimmers. Acta Pædiatr.

[36] Courteix D, Obert P, Lecoq A-M, Guenon P, Koch G (1997). Effect of intensive swimming training on lung volumes, airway resistances and on the maximal expiratory flow-volume relationship in prepubertal girls. Eur J Appl Physiol Occupat Physiol.

[37] Boeckh-Behrens WU, Buskies W, Loges D, Winsen L (2002). Gesundheitsorientiertes Fitnesstraining.

[38] Patessio A, Donner CF (1994). Selection criteria for exercise training in patients with COPD. Zeitschrift für Kardiol.

[39] Forte VA, Leith DE, Muza SR, Fulco CS, Cymerman A (1997). Ventilatory capacities at sea level and high altitude. Anglais.

[40] Gautier H, Peslin R, Grassino A, Milic-Emili J, Hannhart B, Powell E (1982). Mechanical properties of the lungs during acclimatization to altitude. J Appl Physiol.

[41] Landstra AM, Postma DS, Boezen HM, Van Aalderen WMC (2002). Role of Serum Cortisol Levels in Children with Asthma. Am J Respir Crit Care Med.

